# The clinical and functional characteristics of bronchiectasis among tuberculosis patients in Upper Egypt: a single-center study

**DOI:** 10.1186/s43168-022-00112-2

**Published:** 2022-03-18

**Authors:** Maiada K. Hashem, Youssef Suliman Marey Nasim, Aliae A.R. Mohamed-Hussein, Ahmad M. Shaddad

**Affiliations:** 1 Chest Department, Faculty of Medicine, Assiut University, Assiut, 71515, Egypt; 2 Assiut Chest Hospital, Assiut, Egypt

**Keywords:** Bronchiectasis, Tuberculosis, Bronchiectasis Severity Index, Disease burden

## Abstract

**Background:**

Tuberculosis (TB) is considered one of the most common causes of bronchiectasis. Bronchiectasis increases clinical and financial burden of patients with TB. Here, we aim to assess the prevalence of bronchiectasis and its characteristics in patients with TB.

**Results:**

Over 1 year duration, 85 patients with confirmed TB were enrolled in the study. Those patients were clinically, laboratory, and radiologically evaluated. Any patient with other chest diseases was excluded from the study. Out of those patients, 19/85 (22.4%) patients had bronchiectasis. It was found that patients with bronchiectasis had higher frequency of urban residence, current cigarette or goza smoking, and diabetes mellitus. Hemoptysis and expectoration were the most frequent symptoms. Pulmonary function tests were significantly impaired in patients with bronchiectasis. Disease burden was significantly higher among bronchiectasis group in form of frequent hospitalization, longer hospital stay, and need of oxygen therapy.

**Conclusion:**

Bronchiectasis is not uncommon among TB patients. Co-existence of bronchiectasis with TB has distinctive clinical, and functional characteristics that increase the burden of the diseases in the form of prolonged hospital stay and higher utilization of antibiotics and oxygen therapy. Smoking in tuberculous patients may is significantly associated with bronchiectasis.

## Introduction

There are many reported causes of bronchiectasis, but post-infectious type especially following tuberculosis (TB) is still the main etiology. Yet, in some cases of bronchiectasis the etiology remains obscure and hence, it is named as idiopathic type. It was previously reported that TB patients had a frequency of bronchiectasis that ranges between 19% and 65% with higher incidence in fibroid stage of the disease [1, 2]. However, the relation between tuberculosis and bronchiectasis was historically recognized [3].

Both tuberculosis and bronchiectasis are associated with chronic progressive deterioration in pulmonary function which can be presented by symptoms such as cough, wheezes, and dyspnea causes variable degrees of disabilities [4]. Moreover, the risk of hospitalization in cases of TB with bronchiectasis is high [5]. So, early detection of TB patients with subsequent prompt therapy may be associated with reduction in number of cases with bronchiectasis and its complications.

In Egypt, the prevalence and burden of TB is well studied but there is paucity in data about bronchiectasis (prevalence, patterns, and burden) in TB patients. This study aims to assess the prevalence of bronchiectasis among TB patients in our locality and to study its clinical functional characteristics.

## Patients and methods

### Study design and setting

A cross-sectional study was carried out over 1 year duration between January 2020 and December 2020. It was conducted at Outpatient Clinic of Assiut Chest Hospital.

### Inclusion criteria

All patients above 18 years old of both genders with confirmed pulmonary TB attended the outpatient clinic during the study period were recruited. All included patients had finished the initial anti-tuberculosis treatment and converted to sputum negative regardless their regimen.

### Exclusion criteria

Any patient with one or more of the following criteria was excluded; age < 18 years old, extrapulmonary TB, other pulmonary diseases, pregnant women, and/or patient’s rejection to participate.

### Participants

**Fig. 1:**
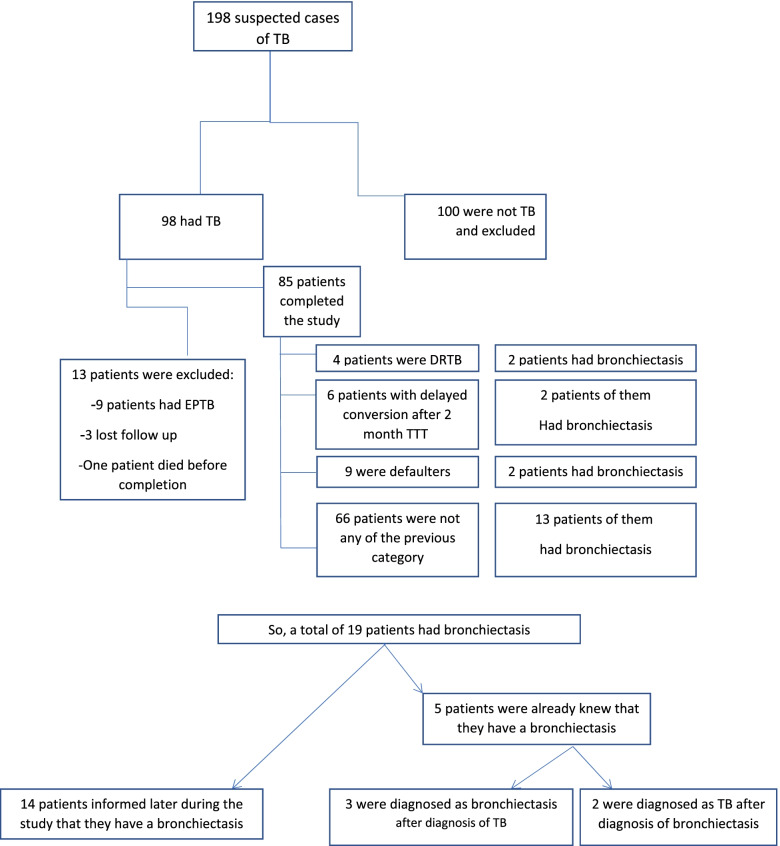
Flow chart of the tuberculosis patients included in the current study. *TB* tuberculosis, *DRTB* drug-resistant tuberculosis, and *EPTB* extrapulmonary tuberculosis

During the study period, 198 patients were suspected of having TB. Only 98 patients had confirmed TB while the other patients were negative and excluded from the study. Other nine patients were excluded from the study because they had extrapulmonary TB. A total of 89 patients with pulmonary TB were recruited where one patient was dead, and three patients were lost before completing the study. Thus, a total of 85 patients were enrolled, completed, and eligible for the analysis in the study. Out of those 85 patients; 19 (22.4%) patients had bronchiectasis. Five patients out of the 19 bronchiectasis patients were diagnosed with bronchiectasis before or at the time of TB diagnosis so they considered as pre-existing bronchiectasis in TB patients while the other 14 patients were diagnosed as post-TB bronchiectasis (Fig. 1).

Bronchiectasis was primary diagnosed based on high-resolution computed tomography (HRCT) chest findings and clinical judgment. Post-TB bronchiectasis was suggested if history, or clinical evidence of TB was present and radiological findings of bronchiectasis in the same lung zone previously affected by TB [6]. On the other hand, patients with history and radiological evidence of bronchiectasis preceding the diagnosis of TB were included as pre-existing of bronchiectasis with tuberculosis.

According to the presence or absence of bronchiectasis, the patients were classified into 2 groups: Group I included tuberculosis patients with bronchiectasis (*n*=19) and Group II included tuberculosis patients without bronchiectasis (*n*=66). The two groups were compared regarding baseline and demographic data, symptoms, pulmonary function tests, and disease burden in the form of frequency of outpatient clinic visits or hospitalization, need for antibiotics, and length of hospital stay.

### Methodology

History taking (with especial emphasis on previous history of TB and received therapy and its duration) with full clinical interpretation were done. Clinical presentation, smoking status, and comorbidities were recorded.

All patients were subjected to routine laboratory investigation including complete blood picture, coagulation profile, and serum creatinine. Plain chest radiogram was done in addition to HRCT to confirm the diagnosis. Pulmonary function was assessed in all participants after conversion to sputum negative.

### Bacteriological evaluation

Bacteriological evaluation of those patients was done by (1) direct smear for acid fast bacilli (at least 3 consecutive sputum samples were collected); (2) Xpert MTB/RIF assay that was done by gene Xpert diagnostic system, Ceipheid Inc., with 4 modules made in USA; and (3) TB culture where the bottles of Lowenstein Jonson media were inoculated with prepared samples then incubated in horizontal position for 2448 h continued for 8 weeks and inspected weekly. Culture was considered positive if at least 50 colonies were presented. Four (4.7%) out of the 85 included patients had rifampicin resistant TB by gene Xpert (Fig. 1).

### Radiological evaluation

**Plain chest radiograph** was done by Genesis X-ray machine (2D), code MI, Switzerland. TB findings were classified into either mild (unilateral cavity or pleural effusion), moderate (bilateral cavity and/or unilateral lung tissue destruction), or sever (bilateral lung tissue destruction) [7].

In case of bronchiectasis, it may be normal but some cases may have enlarged peripheral cysts and mild calcification of hilar mediastinal lymphadenopathy [8, 9].

**High-resolution computed tomography (HRCT) chest** was conducted with Toshiba 16, model name Alexion device. Air space consolidation is the typical findings in case of pulmonary TB. Solitary cavity lesion may be present in 10% of patients. Other less frequent findings included relatively poor defined per bronchial nodules, acinar shadows, large lobular consolidation, and/or multiple cavitary lesions.

In case of bronchiectasis, the following findings may be present; proximal and distal wall thickening, proximal dilatation, distal bronchial dilatation, and tree in bud pattern. Based on HRCT, bronchiectasis could be classified into tubular, cylindrical (abnormal dilatation of bronchus with tram track sign) and cystic (saccular dilatation with balloon cut line traced to pleura), and varicose which has no regular size or pattern with irregular bulge.

### Assessment severity of bronchiectasis

Bronchiectasis Severity Index (BSI) and Fev1/Age/Colonization/Extension/Dyspnea score for bronchiectasis severity (FACED) scores were used to assess severity of bronchiectasis in the current study.

BSI is calculated based on age, body mass index (BMI), predicted forced expiratory volume in 1 s (FEV1)%, history of respiratory hospitalization in the preceding 2 years, number of exacerbations in the previous year, Medical Research Council (MRC) Breathlessness Score, Pseudomonas colonization with other organisms (detected by bacteriological culture and sensitivity tests), and radiological extension of the disease based on HRCT findings [10].

The FACED score is calculated based on age, predicted FEV1%, MRC Breathlessness Score, Pseudomonas colonization, and radiological extension of the disease based on HRCT findings [11].

### Statistical analysis

Data analysis was performed using the software SPSS (Statistical Package for the Social Sciences) version 20. Quantitative variables were described using their means and standard deviations. Categorical variables were described using their absolute frequencies and were compared using chi-square test when appropriate.

Kolmogorov-Smirnov (distribution-type) test was used to verify assumptions for use in parametric tests. To compare continuous data between two continuous variables, independent sample test (for parametric data) and Mann-Whitney test (for non-parametric data) were used. To compare quantitative continuous data between more than two groups, Kruskal-Wallis (KW) test was used when data is not normally distributed, and pairwise comparison was used when the difference is significant in KW analysis to identify group responsible for significant difference. The level of statistical significance was set at *P*<0.05.

## Results

Eighty-five pulmonary tuberculosis patients were enrolled in this study, among them, 19 (22.4%) patients had bronchiectasis. It was found that the majority (78.9%) of patients with bronchiectasis came from urban areas. Also 84.2% of those patients had low socioeconomic class. Patients with bronchiectasis had significantly higher current cigarette smoking (15 (78.9%) vs. 16 (24.2%); *p*= 0.001), goza smoking (13 (68.4%) vs. 17 (25.8%); *p*< 0.001), diabetes mellitus (11 (57.9%) vs. 15 (22.7%); *p*= 0.003), and neurological disease (7 (36.8%) vs. 6 (9%); *p*= 0.007) (Table 1).

**Table 1:** Baseline data of patients with TB based on the presence of bronchiectasis (*N*=85)

Parameter	Total ***N***=85	Bronchiectasis		***P*** value
		Present ***N***=19	Absent ***N***=66	
**Age**	46.81 ±8.42	45.53±9.74	47.18±8.05	0.273
**Gender**				0.896
Male	68 (80.0)	15 (78.9)	53 (80.3)	
Female	17 (20.0)	4 (21.1)	13 (19.7)	
**Residence**				0.001
Rural	46 (54.1)	4 (21.1)	42 (63.6)	
Urban	39 (45.9)	15 (78.9)	14 (36.4)	
**Occupation**				0.333
Housewife/not working	19(22.4)	3 (15.8)	16 (24.2)	
Unskilled worker	32(37.6)	5 (26.3)	27 (40.9)	
Skilled worker	18(21.2)	6 (31.6)	12 (18.2)	
Clerk/free trade	9(10.6)	2 (10.5)	7 (10.6)	
Semiprofessional/professional	7(8.2)	3 (15.8)	4 (6.1)	
**Social class**				0.004
Low	43(50.6)	16 (84.2)	27 (40.9)	
Middle	30(35.3)	2 (10.5)	28 (42.4)	
High	12(14.1)	1 (5.3)	11 (16.7)	
**Marital status**				0.294
Single	9(10.6)	2 (10.5)	7 (10.6)	
Married	59(69.4)	11 (57.9)	48 (72.7)	
Divorced/widow	17(20)	6 (31.6)	11 (16.7)	
**Current cigarette smoking**	31(36.5)	15 (78.9)	16 (24.2)	<0.001
**Goza smoking**	30(35.3)	13 (68.4)	17 (25.8)	0.001
**Drug addict**	18(21.21)	6 (31.6)	12 (18.2)	0.208
**Comorbidities**				
Diabetes mellitus	26 (30.6)	11 (57.9)	15 (22.7)	0.003
Hypertension	44 (51.8)	12 (63.2)	32 (48.5)	0.259
Liver disease	30 (35.3)	9 (47.4)	21 (31.8)	0.211
Kidney disease	24 (28.2)	4 (21.1)	20 (30.3)	0.567
Cardiac disease	17 (20)	6 (31.6)	11 (16.7)	0.152
Neurological disease	13 (15.3)	7 (36.8)	6 (9)	0.007

Data expressed as mean (SD), frequency (percentage). *P* value was significant if < 0.05. TB: tuberculosis

**Fig. 2:**
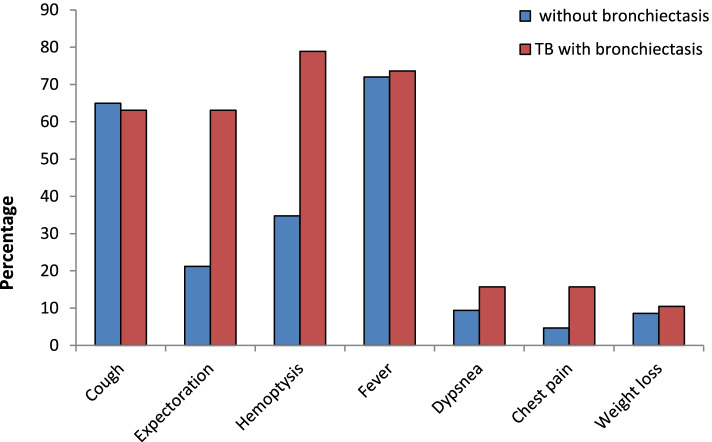
Clinical presentation among enrolled TB patients based on the presence of bronchiectasis (*N*=85)

The most common symptoms in bronchiectasis patients were expectoration and hemoptysis while compared to tuberculosis patients without bronchiectasis who complained mainly of cough without expectoration and fever. Less common-reported symptoms were dyspnea and chest pain, which occurred during acute exacerbations (Fig. 2).

**Fig. 3:**
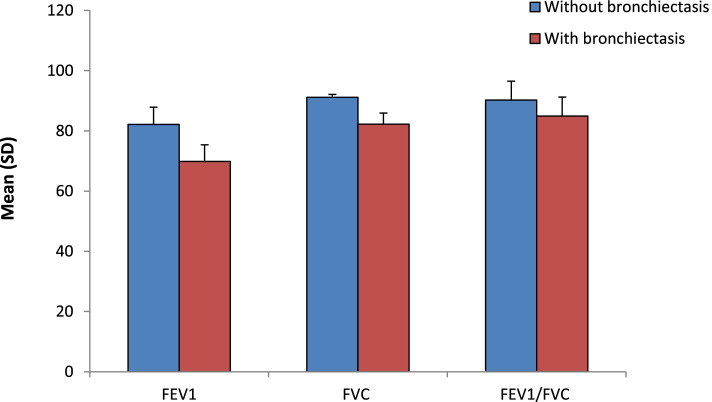
Pulmonary function test among studied TB patients (after conversion to sputum negative) based on the presence of bronchiectasis (*N*=85). *FEV1* forced expiratory volume in 1st second; *FVC* forced vital capacity

Patients with bronchiectasis had worth spirometry results. There was statistically significant difference in pulmonary function test in both groups; FEV1, FVC, and FEV1/FVC ratio were all significantly lower in patients with bronchiectasis (Fig. 3).

Moreover, as shown in Table 2, patients with bronchiectasis represented higher disease burden. There was statistically significant difference between the studied groups regarding frequency of hospitalization, length of hospital stays, frequency of outpatient visits, days of antibiotic use over the year, and need of oxygen therapy. All were significantly higher in patients with bronchiectasis. However, regarding response to treatment, 10.5% of patient with bronchiectasis had delayed conversion to sputum negative compared to 6.1% in patients without bronchiectasis with no statistically significant difference. Also, there was no significant difference in the need for shift to 18 months regimen between both groups.

**Table 2:** Disease burden in patients with TB based on presence of bronchiectasis (*N*=85)

	Bronchiectasis		
	Present (***n***= 19)	Absent (***n***= 66)	***P*** value
**Frequency of hospitalization**	2 (1–2)	1 (0–1)	0.007
**Length of hospital stay (day)**	10 (4–15)	3 (0–7)	<0.001
**Frequency of outpatient visit**	8 (5–8)	3 (3–4)	<0.001
**Days with antibiotics**	50 (45–60)	28 (20–35)	<0.001
**Need for oxygen therapy**	10 (52.6)	10 (15.2)	<0.001
**TB treatment outcome,** ***N*** **(%)**			>0.999
Defaulters	2 (10.5)	7 (10.6)	0.612
Delayed conversion	2 (10.5)	4 (6.1)	0.215
Shift from 6- to 18-month regimen	2 (10.5)	2 (3)	

Data expressed as frequency (percentage) and median (range). *P* value was significant if < 0.05

According to BSI score 42.1%, 15.8%, and 42.1% of patients with bronchiectasis had low, intermediate, and high disease severity, respectively. Table 3 demonstrates the statistically significant association between BSI score and frequency of outpatient clinic visits. On pairwise comparison, the difference was significant between intermediate and high groups. However, there was non-significant association between BSI score and either frequency of hospitalization, length of hospital stays, and need of oxygen therapy or days with antibiotic use.

**Table 3:** Relation between BSI and burden of bronchiectasis (*n*=19)

Parameter	BSI			
	Low	Intermediate	High	***P***
**Frequency of hospitalization**	1.5 (0.25–2)	1 (0–1)	2(1.25–3)	0.115
**Length of hospital stay (day)**	4.5 (4–12.5)	10 (0–10)	11(9.25–17)	0.1
**Frequency of outpatient visit**	6 (5–8)	12 (5–12)	9.5 (6.75–12)	0.043
**Pairwise**	**P**_**1**_ **0.194**	**P**_**2**_ **0.01**	**P**_**3**_ **0.756**	
**Days with antibiotics**	47.5 (45–72.5)	45 (40–45)	60 (52.5–67.5)	0.299
**Need for O**_**2**_	3 (37.5)	2 (66.7)	5 (62.5)	0.566

Data expressed as frequency (percentage) and median (range). *P* value was significant if < 0.05. *BSI* bronchiectasis severity index

p1 the difference between low and intermediate; p2 the difference between moderate and high; p3 the difference between low and high

On the other hand, regarding FACED score, 31.6% of bronchiectasis patients had mild, 31.6% moderate, and 36.8% severe disease. Table 4 demonstrates the statistically significant association between FACED score and frequency of hospitalization. On pairwise comparison, the difference was significant between severe and each other group (highest in those with severe score). There was statistically significant association between FACED score and length of hospital stay. On pairwise comparison, the difference was significant between severe and moderate groups.

**Table 4:** Relation between FACED and burden of bronchiectasis (*N*=19)

Parameter	FACED			
	Mild	Moderate	Severe	***P***
**Frequency of hospitalization**	1 (0.75–2)	1.5 (0–2)	2 (2–3)	0.049
**Pairwise comparison**	**P**_**1**_ **0.932**	**P**_**2**_ **0.049**	**P**_**3**_ **0.03**	
**Length of hospital stay (day)**	3 (10–16)	4.5 (3–8.5)	12 (10–17)	0.043
**Pairwise comparison**	**P**_**1**_ **0.619**	**P**_**2**_ **0.036**	**P**_**3**_ **0.694**	
**Frequency of outpatient visit**	6.5 (8–10.75)	5 (5–7)	10 (9–12)	0.036
**Pairwise**	**P**_**1**_ **0.581**	**P**_**2**_ **0.03**	**P**_**3**_ **0.656**	
**Days with antibiotics**	52.5 (45–72.5)	47.5 (37.5–50)	60 (60–70)	0.091
**Need for O**_**2**_	3 (50)	2 (33.3)	5 (71.4)	0.455

Data expressed as frequency (percentage) and median (range). *P* value was significant if < 0.05. *FACED* Fev1/Age/Colonization/Extension/Dyspnea score for bronchiectasis severity

p1 the difference between low and intermediate; p2 the difference between moderate and high; and p3 the difference between low and high

There was statistically significant association between FACED score and frequency of outpatient clinic visits. On pairwise comparison, the difference was significant between severe and moderate groups. There is statistically non-significant association between FACED score and duration of antibiotic use during the previous year or need of oxygen.

## Discussion

Consistent with the high prevalence of tuberculosis in developing countries TB was the most frequent underlying cause of bronchiectasis, and when combined with other severe infections, it accounted for 58% of all cases of bronchiectasis [12].

Egypt is a middle/low TB burden country. Egyptian Ministry of Health and Population adopted the latest National Tuberculosis Control Program since September 2007 and showed progressive decrease in the incidence of TB from 21/100 000 populations in 2006 to 13/100 000 populations in 2017, [13] then incidence decreased again to be 12/100000 population in the last WHO estimation of burden of TB in Egypt in 2019 [14].

Based on the current study, bronchiectasis was found in only 19 (22.4%) patients. Among them, 5 patients diagnosed with bronchiectasis before or during TB diagnosis.

The prevalence of bronchiectasis in TB patients varies widely in literatures. Jin et al. (2018) concluded that frequency of bronchiectasis was 64.4% in patients with TB. Also, the authors said that previous pulmonary TB was an independent risk factor for coexistent bronchiectasis in COPD, suggesting that TB might be a cause of bronchiectasis in these patients [15]. In another Chinese study, post-tuberculosis was the second common cause of bronchiectasis with (16.0%) prevalence [16]. Nevertheless, other studies in European and American populations found that post-tuberculosis bronchiectasis only accounted for 2–6.3% [17, 18].

A previous Egyptian study concluded that the prevalence of bronchiectasis was 47.8%; it was primarily of cylindrical type, mainly localized in the lower lobes. The difference between this study and ours may be attributed to patients’ selection where they enrolled only patients with COPD [19].

In light of the forementioned data, tuberculosis is still an important cause of bronchiectasis in developing countries. According to the 2014 WHO Global tuberculosis report, 44% and 22% of new tuberculosis cases occur in Asian and African countries, respectively [20].

In the current study, 85 patients with pulmonary TB were recruited. The mean age of those patients was 46.81 years with range between 30 and 62 years. In consistent with the current study, Malik et al. (2021) stated that the majority of cases with TB occurred between 18 and 65 years [21]. Unlike Yang et al. (2019) who reported that bronchiectasis was more prevalent at the age of 40 or older [22], it was found that age of those with bronchiectasis was insignificantly lower than those without bronchiectasis.

In this study, patients with bronchiectasis reported higher frequency of goza and cigarette smoking in comparison to those without bronchiectasis. In contrast, Yang et al. (2019) found that bronchiectasis was common in non-smokers and females. These differences could be explained by different population, selection bias, and sample size [22].

In addition, low socioeconomic status was associated with bronchiectasis in this study. This could provide informative data that establish the association between socioeconomic status and bronchiectasis. Low family income, limited physical activity, and presence of respiratory symptoms were reported as predictors of bronchiectasis [22].

Diabetes mellitus was frequently present among patients with bronchiectasis in the current study. In contrast, Dou et al. (2018) found that diabetes mellitus and hypertension showed no significant difference between those with bronchiectasis and those without. The association between diabetes mellitus and bronchiectasis is not fully studied, and so, future studies about this point are highly warranted [23].

Hemoptysis was the most frequent symptom reported by bronchiectasis group of patients recruited in this study followed by fever, cough, and expectoration with significant difference compared to non-bronchiectasis tuberculosis group who complained mainly of fever and cough without expectoration. Hemoptysis was also more common in post-tuberculosis bronchiectasis in Chinese population [24]. This could be explained by the pathological changes associated with bronchiectasis including permanent destruction of airways that predisposes to lifelong morbidity with recurrent episodes of infections, purulent sputum production, hemoptysis, and sometimes progression to pneumonia [25].

Regarding change in pulmonary function tests, we found that FEV1, FVC, and FEV1/FVC were significantly lower among patients with bronchiectasis. This pattern of pulmonary function was consistent with a previous study that found a reduced FEV1, FVC, and FEV1/FVC among patients with bronchiectasis in comparison to control subjects [22]. Chronic inflammatory response and long-term anatomic alterations induced by TB were believed to be the main pathological basis for the impairment of lung function and poor prognosis with subsequent TB-associated structural alterations, such as destruction of elastic and muscular components of the bronchial walls, scar formation, bronchial stenosis, and bronchiectasis which is associated with airflow limitation [26]. Furthermore, in a study following the progressive changes in lung structure and pulmonary function in patients with TB, patients with cavities had significantly lower FEV1 at baseline and after 1 month of TB treatment initiation compared to patients without cavities [27].

In the current study, patients with bronchiectasis had significantly higher frequency of oxygen therapy utilization, hospitalization, and visiting of the outpatient clinic. Also, those patients had longer hospital stay. It is known that bronchiectasis is associated with markedly increased healthcare costs, frequent hospitalization, and mortality [28, 29]. Although we were unable to calculate the exact associated costs for the in-patient care of bronchiectasis patients, our data on the mean hospital stay emphasize that bronchiectasis may account for a significant, though underappreciated economic burden in healthcare.

On the other hand, our study did not demonstrate either the impact of presence of bronchiectasis tuberculosis treatment results or the impact of DRTB and treatment failure on bronchiectasis development as there was no significant difference between both groups. This could be attributed to the small number of patients who were DRTB, defaulters, delayed conversion, or shifted to 18 months regimen. Only 4/85 (4.7%) patients had DRTB. It was found that those patients had significantly younger age in comparison to those without DRTB (32.5 ± 1.73 vs. 47.52 ± 7.98 years; *p*< 0.001) and all of them had liver disease. This study had high percentage of DRTB in comparison to Abd El Malik et al. (2021) [21] who found out 20/3357 (0.59%) patients had DRTB. Moreover, there was no statistical difference between DRTB prevalence in patients with and without bronchiectasis. This could be attributed to small sample size in the current study. However, in this respect, it is important to note that the presence of bronchiectasis is increasingly recognized to be associated with a poorer overall prognosis among such patients, as very recently demonstrated by a previous study [30, 31].

Bronchiectasis severity assessment is crucial to plan good bronchiectasis management. Both BSI and FACED scoring systems include FEV1, P. aeruginosa colonization, HRCT, and dyspnea scores. Nonetheless, the disparity in the remaining components would still contribute to the differences in how these two scores reflect disease severity. BSI was found to be sensitive tool in predicting annual risks of hospitalization and mortality. On the other hand, FACED score predicted long-term mortality well and that FACED appeared to be simpler [32]. In the current study 42.1% of bronchiectasis patients had high severity by BSI and 36.8% were sever by FACED score. Thus, there was significant substantial agreement between the two scores. Unlike the abovementioned review, we reported significant association between FACED score in severe and moderate patients and frequency of hospitalization, length of hospital stay, and frequency of outpatient clinic visits. Thus, increased disease severity might increase its burden. However, we could not rely on these results due to limited recruited subjects.

The main limitations of the current study were (1) the cross-sectional nature of the study which did not allow longer follow-up for included patients. Thus, long-term effect of TB on the structure and function of the lung was not evaluated. (2) The small sample size, especially for DRTB patients, defaulters, and treatment failures, did not give us a true image about the impact of TB treatment on bronchiectasis development. (3) The social and economic burden of bronchiectasis on TB patients were not addressed. (4) The study discussed the characteristic features of bronchiectasis in TB patients but did not demarcate the difference between TB patients with preexisting bronchiectasis and those with post-TB bronchiectasis due to small number of patients recruited. (5) It was a single-center study; further national wide studies are needed to estimate the actual prevalence of the disease. (6) The emergence of coronavirus disease 2019 pandemic, with its consequences, during the study period hampered patients, healthcare providers, and researchers attending TB management facilities with its impact on such studies.

In conclusion, bronchiectasis is not uncommon among TB patients in our community. It is more common in lower socioeconomic status, cigarette, and goza smokers and in patients with diabetes mellitus. Co-existence of bronchiectasis with TB has distinctive clinical and functional characteristics in the form of increased expectoration, hemoptysis, and decreased FEV1. Bronchiectasis may increase medical demands in patients with tuberculosis namely antibiotics and oxygen therapy utilization, outpatient clinic visits, and prolonged hospitalization. Further studies with larger sample size are required to assess the relation between DRTB and bronchiectasis.

## Data Availability

The datasets analyzed during the current study are available upon request.

## Abbreviations

BMI: Body mass index
BSI: Bronchiectasis Severity Index
DRTB: Drug-resistant tuberculosis
FACED: FEV1/Age/Colonization/Extension/Dyspnea Score
FEV1: Forced Expiratory Volume in One Second
FVC: Forced vital capacity
HRCT: High-resolution computed tomography
MRC: Medical research council
TB: Tuberculosis
